# Sleep Disorders Following Mild and Moderate Traumatic Brain Injury

**DOI:** 10.3390/brainsci9010010

**Published:** 2019-01-11

**Authors:** Laith Thamer Al-Ameri, Talib Saddam Mohsin, Ali Tarik Abdul Wahid

**Affiliations:** 1 Al-Kindy College of Medicine, University of Baghdad, Baghdad, Iraq; talib.almuhsin@gmail.com; 2 College of Medicine, University of Baghdad, Baghdad, Iraq; azdh_1978@yahoo.com

**Keywords:** traumatic brain injury, sleep disorders, sleep latency

## Abstract

(1) Background: Sleeping disorders are frequently reported following traumatic brain injury (TBI). Different forms of sleeping disorders have been reported, such as sleepiness, insomnia, changes in sleeping latency, and others. (2) Methods: A case-control study with 62 patients who were victims of mild or moderate TBI with previous admissions to Iraqi tertiary neurosurgical centers were enrolled as the first group, and 158 patients with no history of trauma were considered as the control. All were 18 years of age or older, and the severity of the trauma and sleep disorders was assessed. The Pittsburgh sleep quality index was used to assess sleep disorders with average need for sleep per day and average sleep latency were assessed in both groups. Chi-square and t-test calculations were used to compare different variables. (3) Results: 39 patients (24.7%) of the controlled group experienced sleeping disorders compared to TBI group with 45 patients (72.6%), *P*-value < 0.00001. A total of 42 patients were diagnosed on admission as having a mild degree of TBI (mean GCS 13.22 ± 1.76) and 20 patients were diagnosed with moderate TBI (mean GCS11.05 ± 1.14. 27). A total of 27 (46.28%) patients with mild severity TBI and 18 patients (90%) of moderate severity were considered to experience sleeping disorders, *P*-value 0.0339. Each of the mild and moderate TBI subgroups show a *P*-value < 0.00001 compared to the control group. Average sleep hours needed per day for TBI and the control were 8.02 ± 1.04 h and 7.26 ± 0.58 h, respectively, *P*-value < 0.00001. Average sleep latency for the TBI and the control groups were 13.32 ± 3.16 min and 13.93 ± 3.07 min respectively, *P*-value 0.065. (4) Conclusion: Sleep disturbances are more common following mild and moderate TBI three months after the injury with more hours needed for sleep per day and no significant difference in sleep latency. Sleep disturbances increase in frequency with the increase in the severity of TBI.

## 1. Introduction

Sleep disorders frequently follow a traumatic brain injury (TBI), with reports of 30%–70% of TBI patients experiencing negative impacts on quality of life and rehabilitation [1,2,3,4,5]. Sleep disorders have been shown to aggravate psychiatric problems, affect the mood and behavior of injured patients, and thus contribute to poor neuronal remodeling following the injury [6,7]. Different patterns of sleep disorders have been reported, such as hypersomnia, insomnia, change in sleep latency, narcolepsy, and parasomnia. [4,6,7,8].

Many factors contribute to sleep disorders, with brain trauma itself being the most important, including primary and secondary effects. A primary effect results from direct injury to brain tissues through acceleration-deceleration forces and/or rotational forces with consequent diffuse axonal injury, while secondary effects results from cellular events caused by hypoxia and raised intracranial pressure [9]. 

Specific locations of brain injury may be responsible for specific patterns of sleeping disorders. Thalamic and caudal midbrain injury may be responsible for hypersomnia, while direct injury impact on the pineal gland and tentorium may result in insomnia due to the effect on melatonin homeostasis [2,4,10,11,12].

Another hypothesis states that supra-chiasmatic damage may result in a consequent disturbance in the circadian rhythm, with a mixed picture of insomnia and hypersomnia [4].

Rather than factors related to brain trauma itself, sleep disorders may be a result of neuropsychiatric conditions, such as anxiety or depression, or a result of pain due to associated musculoskeletal trauma or spasm. Anxiety, depression, and pain are considered to be comorbidity factors associated with sleep disorders following TBI [4,13,14]. 

Traumatic brain injury is a leading cause of death and disability, with different etiologies, types, and severity. Different patterns of complications and outcomes vary widely according to the severity of TBI [15,16]. The severity is assessed by the Glasgow Coma Scale (GCS) based on the level of consciousness, with the TBI being classified into mild (GCS 15-13), moderate (12-9), or severe (8 or less) [15,17]. 

The Pittsburgh sleep quality index (PSQI) is used to assess sleep disorders and shows good reliability and validity in both young and old age groups [18,19,20]. The PSQI has been translated into the Arabic language successfully, showing acceptable reliability and validity [21].

Our study aimed to evaluate sleep disorders in patients with previous mild and moderate TBI that were admitted to tertiary neurosurgical centers in Baghdad, Iraq. It is one of the first studies conducted on patients attending Iraqi tertiary neurosurgical centers and clinic to consult for such a reason.

## 2. Materials and Methods

This is a case-control study with 220 patients enrolled; 62 of them were victims of mild or moderate TBI (three months post-injury) who previously attended tertiary neurosurgical centers in Baghdad, Iraq, and 158 patients had no history regarding TBI, considered to be the control group. The TBI patients were with different etiologies, including falling from a height, road traffic accidents, and being hit by an object, and all presented with no persistent neurological deficit. Age and sex was matched between both groups. Of the TBI group, 42 out of 62 suffered a mild TBI while the other 20 patients were considered to have moderate TBI. Severity determination of the TBI was based on assessment through the GCS. All participants in both groups were 18 years or older. Patients of the TBI group with sleeping disorders or psychiatric disorders prior to the injury were excluded from the study. The exclusion criteria of the control group were patients with psychiatric illnesses, previous TBI, or those who were on hypnotics.

Admission GCS scores were obtained from the patients’ records, while sleep disorders were assessed through the PSQI translated to the Arabic language for self-reporting sleep quality. The PSQI were administered to patients three months following the TBI. Global scoring was undertaken for patients in both groups to assess the presence of sleep disorder, with the assessment including the average need for sleep per day and average sleep latency. The global PSQI score is calculated by totaling the seven component scores, providing an overall score ranging from 0 to 21, where a lower score denotes a healthier sleep quality. Traditionally, the items from the PSQI have been summed to create a total score to measure overall sleep quality. A total score of 5 or greater indicates poor sleep quality, and a score lower than 5 is regarded as normal.

Statistical analysis was completed with data introduced to IBM SPSS 22 software (Chicago, IL, USA). Chi-square and *t*-test calculations were used to compare different variables with a *P*-value ˂ 0.05 considered significant. Normality of distribution of dependent variables was checked through application of Shapiro–Wilk, Levene’s test was used to find out homogeneity of dependent variables variances.

Ethical and scientific approval was obtained from the scientific unit at Al-Kindy College of Medicine, University of Baghdad. Written consent was obtained from each participant enrolled in the study after clarification to them of the purpose of the study.

## 3. Results

Data was collected from both groups and analyzed using a Chi-square and *t*-test. The male to female ratio was 4:1, with a mean age of 35 ± 14 years. Mean age for TBI group was 36.3 ± 14.94, while mean age for control group was 34.59 ± 13.8, see Table 1 and Table 2. A total of 39 patients (24.7%) of the control group experienced sleeping disorders according to the PSQI, and 119 did not, while 45 patients (72.6%) of the TBI group experienced sleeping disorders and 17 patients did not. *P*-value was <0.00001, see Table 3.

Of the TBI group, 42 patients were diagnosed on admission as having a mild degree of TBI, with a mean GCS score of 13.22 ± 1.76. A total of 20 patients were diagnosed as having a moderate TBI, with a mean GCS score of 11.05 ± 1.14. Both subgroups were triaged as having TBI by a neurosurgical on call trauma team. Of these subgroups, 27 patients (46.28%) of mild severity TBI and 18 patients (90%) of moderate severity experienced sleeping disorders according to the PSQI. Comparing both subgroups (mild and moderate TBI) showed a *P*-value of 0.0339. Each of mild and moderate TBI subgroups showed a *P*-value <0.00001 compared to the control group, see Table 4.

The average sleep hours needed per day was assessed for patients in the TBI and control groups, with means of 8.02 ± 1.04 h and 7.26 ± 0.58 h, respectively. *P*-value < 0.00001, Table 3.

The average sleep latency was assessed for patients in the TBI and control groups, with means of 13.32 ± 3.16 min and 13.93 ± 3.07 min, respectively. *P*-value 0.065, Table 3.

## 4. Discussion

Sleep disturbances were more common in the TBI group than the control group, with a highly significant *P*-value ˂ 0.00001. A total of 72.6% of the TBI group experienced sleep disturbances, compared to 24.7% in the control group. Sleep disturbances following TBI have been reported frequently in many previous studies when compared to controls. Sleep disturbances in previous studies have, however, shown different pictures. Baumann et al. in 2007 showed 72% of people experienced sleep disturbances following a TBI [6]. Others showed rates that varied widely from 30% to 84% [22]. The extremely high rate obtained in our results may be explained by poor rehabilitation following TBIs in our area. 

Different pictures were also shown according to the severity of TBI. Our study assessed sleep disturbances in both mild and moderate groups, separately. The mild TBI and moderate TBI groups showed 46.28% and 90% sleep disturbances rates, respectively. Both had a highly significant *P*-value compared to the control groups (*P*-value ˂ 0.00001); the moderate TBI group had a higher rate than the mild TBI group, with a significant *P*-value of 0.0339. These results are in agreement with the hypothesis stating that sleep disturbances are caused by neuronal damage, such as the damage caused by primary or secondary brain injuries leading to diffuse axonal injury affecting sleep-regulating regions. This type of damage is expected to be greater the more severe the TBI is [4,5]. 

Our study shows that more hours are needed for sleep per day in the TBI group compared with the control group, with a mean average hours of 8.02 ± 1.04 and 7.26 ± 0.58, respectively. The *P*-value was highly significant (*P* ˂ 0.00001). These results agree with others, including Lukas et al. who showed similar result of 8.3 ± 1.1 versus 7.1 ± 0.8 h for the TBI group and the control, respectively, with high significance (*P* ˂ 0.00001) [5]. 

Concerning sleep latency, although the TBI group was lower than the control group at 13.93 ± 3.07 and 13.22 ± 3.16, respectively, the difference was not significant between the TBI and control groups, with a *P*-value of 0.065. Lukas et al. demonstrate significant difference in sleep latency, with a *P*-value of 0.0009 [5]. However, in their meta-analysis, Grima et al. [3] showed no difference in sleep latency when comparing different studies. They did find shorter sleep latency to REM sleep, however this was not analyzed in our study due to laboratory limitations. 

Limitations of the study include small sample size, the fact that patients were from centers in Baghdad City only, and the unavailability of well-equipped sleep laboratories. Further studies are needed to include all Iraqi governorates.

## 5. Conclusions

Sleep disturbances are common in patients following mild and moderate TBI three months after the injury. More hours are needed for sleep per day in previous TBI patients versus the general population. There is no significant change in sleep latency. Sleep disturbances increase in frequency with the increase in the severity of the TBI assessed by the GCS. 

## Figures and Tables

**Table 1:** Difference between mean and standard deviation of age of studied personnel according to the groups.

Group	*N*	Mean Age	Standard Deviation	*P*-Value
Traumatic brain injury (TBI) group	62	36.3065	14.94755	0.418
Control	158	34.5886	13.80950	

**Table 2:** Difference between mean and standard deviation of gender of studied personnel.

Gender	*N*	Mean	Standard Deviation	*P*-Value
male	165	1.7212	0.44977	0.863
female	55	1.7091	0.45837	

**Table 3:** Data for traumatic brain injury (TBI) and the control groups.

	TBI	Control	*P*-Value	Test Value	*df*	Method
**Sleep disorders** **(No. of patients)**	45 (72.6%)	39 (24.7%)	*P* ˂ 0.0001	43.278 χ^2^ value		Chi-square
Sleep hours/day *	7. 94± 1.07	7.26 ± 0.58	*P* ˂ 0.0001	4.72 *t* value	218	*t*-test
Sleep latency (min) *	13.22 ± 3.16	13.93 ± 3.07	*P* 0.131	1.517 *t* value	218	*t*-test

* mean ± standard deviation.

**Table 4:** Data for mild and moderate traumatic brain injury (TBI) subgroups.

	Mild TBI	Moderate TBI	*P*-Value	Method
Initial GCS	13.22 ± 1.76	11.05 ± 1.14		
Sleep disorders (No. of patients)	27 (64.28%)	18 (90%)	0.0339 χ^2^ 4.501	Chi-square

## References

[1] Ponsford J., Parcell D., Sinclair K., Roper M., Rajaratnam S. (2013). Changes in Sleep Patterns Following Traumatic Brain Injury. Neurorehabil. Neural. Repair..

[2] Shekleton J., Parcell D., Redman J., Phipps-Nelson J., Ponsford J., Rajaratnam S. (2010). Sleep disturbance and melatonin levels following traumatic brain injury. Neurology.

[3] Grima N., Ponsford J., Rajaratnam S., Mansfield D., Pase M. (2016). Sleep Disturbances in Traumatic Brain Injury: A Meta-Analysis. J. Clin. Sleep Med..

[4] Viola-Saltzman M., Musleh C. (2016). Traumatic brain injury-induced sleep disorders. Neuropsychiatr. Dis. Treat..

[5] Imbach L.L., Valko P.O., Li T., Maric A., Symeonidou E.R., Stover J.F., Bassetti C.L., Mica L., Werth E., Baumann C.R. (2015). Increased sleep need and daytime sleepiness 6 months after traumatic brain injury: A prospective controlled clinical trial. Brain.

[6] Baumann C., Werth E., Stocker R., Ludwig S., Bassetti C. (2007). Sleep-wake disturbances 6 months after traumatic brain injury: A prospective study. Brain.

[7] Rao V., Rollings P. (2002). Sleep disturbances following traumatic brain injury. Curr. Treat. Opt. Neurol..

[8] Castriotta R., Murthy J. (2011). Sleep Disorders in Patients with Traumatic Brain Injury. CNS Drugs.

[9] Andriessen T., Jacobs B., Vos P. (2010). Clinical characteristics and pathophysiological mechanisms of focal and diffuse traumatic brain injury. J. Cell Mol. Med..

[10] Kemp S., Biswas R., Neumann V., Coughlan A. (2004). The value of melatonin for sleep disorders occurring post-head injury: A pilot RCT. Brain Inj..

[11] Yaeger K., Alhilali L., Fakhran S. (2014). Evaluation of Tentorial Length and Angle in Sleep-Wake Disturbances After Mild Traumatic Brain Injury. AJR Am. J. Roentgenol..

[12] Llompart-Pou J.A., Pérez G., Raurich J.M., Riesco M., Brell M., Ibáñez J., Pérez-Bárcena J., Abadal J.M., Homar J., Burguera B. (2010). Loss of Cortisol Circadian Rhythm in Patients with Traumatic Brain Injury: A Microdialysis Evaluation. Neurocrit. Care.

[13] Viola-Saltzman M., Watson N. (2012). Traumatic Brain Injury and Sleep Disorders. Neurol. Clin..

[14] Wiseman-Hakes C., Murray B.J., Mollayeva T., Gargaro J., Colantonio A. (2015). A Profile of Sleep Architecture and Sleep Disorders in Adults with Chronic Traumatic Brain Injury. J. Sleep Disord. Ther..

[15] Dinsmore J. (2013). Traumatic brain injury: An evidence-based review of management. Continuing Educ. Anaesth. Crit. Care Pain.

[16] Maas A., Stocchetti N., Bullock R. (2008). Moderate and severe traumatic brain injury in adults. Lancet Neurol..

[17] Yates P., Williams W., Harris A., Round A., Jenkins R. (2006). An epidemiological study of head injuries in a UK population attending an emergency department. J. Neurol. Neurosurg. Psychiatry.

[18] Mondal P., Gjevre J.A., Taylor-Gjevre R.M., Lim H.J. (2013). Relationship between the Pittsburgh Sleep Quality Index and the Epworth Sleepiness Scale in a sleep laboratory referral population. Nat. Sci. Sleep.

[19] Spira A., Beaudreau S., Stone K., Kezirian E., Lui L., Redline S., Ancoli-Israel S., Ensrud K., Stewart A. (2011). Reliability and Validity of the Pittsburgh Sleep Quality Index and the Epworth Sleepiness Scale in Older Men. J. Gerontol. A Biol. Sci. Med. Sci..

[20] De la Vega R., Tomé-Pires C., Solé E., Racine M., Castarlenas E., Jensen M., Miró J. (2015). The Pittsburgh Sleep Quality Index: Validity and factor structure in young people. Psychol. Assess..

[21] Suleiman K., Yates B., Berger A., Pozehl B., Meza J. (2009). Translating the Pittsburgh Sleep Quality Index Into Arabic. West J. Nurs. Res..

[22] Zeitzer J., Friedman L., OHara R. (2009). Insomnia in the context of traumatic brain injury. J. Rehabil. Res. Dev..

